# An end-to-end seed vigor prediction model for imbalanced samples using hyperspectral image

**DOI:** 10.3389/fpls.2023.1322391

**Published:** 2023-12-15

**Authors:** Tiantian Pang, Chengcheng Chen, Ronghao Fu, Xianchang Wang, Helong Yu

**Affiliations:** ^1^ College of Computer Science and Technology, Jilin University, Changchun, China; ^2^ Key Laboratory of Symbolic Computation and Knowledge Engineering of Ministry of Education, Jlin University, Changchun, China; ^3^ School of Computer Science, Shenyang Aerospace University, Shenyang, China; ^4^ Chengdu Kestrel Artificial Intelligence Institute, Chengdu, China; ^5^ College of Information Technology, Jilin Agricultural University, Changchun, China

**Keywords:** hyperspectral image, seed vigor prediction, sample imbalance, focal loss, WAResNet, Focal-WAResNet

## Abstract

Hyperspectral imaging is a key technology for non-destructive detection of seed vigor presently due to its capability to capture variations of optical properties in seeds. As the seed vigor data depends on the actual germination rate, it inevitably results in an imbalance between positive and negative samples. Additionally, hyperspectral image (HSI) suffers from feature redundancy and collinearity due to its inclusion of hundreds of wavelengths. It also creates a challenge to extract effective wavelength information in feature selection, however, which limits the ability of deep learning to extract features from HSI and accurately predict seed vigor. Accordingly, in this paper, we proposed a Focal-WAResNet network to predict seed vigor end-to-end, which improves the network performance and feature representation capability, and improves the accuracy of seed vigor prediction. Firstly, the focal loss function is utilized to adjust the loss weights of different sample categories to solve the problem of sample imbalance. Secondly, a WAResNet network is proposed to select characteristic wavelengths and predict seed vigor end-to-end, focusing on wavelengths with higher network weights, which enhance the ability of seed vigor prediction. To validate the effectiveness of this method, this study collected HSI of maize seeds for experimental verification, providing a reference for plant breeding. The experimental results demonstrate a significant improvement in classification performance compared to other state-of-the-art methods, with an accuracy up to 98.48% and an F1 score of 95.9%.

## 1 Introduction

The seed is a vital component of the plant life cycle, containing genetic information and nutrients. It supports plant propagation, survival, adaptability, and dispersal. Healthy and viable seeds are crucial for plant growth and reproduction, increasing plant yields, enhancing plants adaptation to environmental changes, reducing susceptibility to diseases, and contributing to the maintenance of population stability and diversity. By protecting and managing seeds, humanity can preserve and improve plant resources, ensuring food production and ecosystem stability. However, unfavorable conditions such as improper temperature and humidity will lead to the aging and deterioration of seed vigor during storage (Van De Looverbosch et al., 2022). Rapid and accurate identification of seed vigor is essential for improving seed germination rate, increasing plant yield, ensuring product quality and promoting agricultural development. Currently, seed vigor prediction relies on traditional manual inspection, which is non-automated, time-consuming and destructive, requiring specialized training and experienced experts for assessment.

The variations of seed vigor caused by long-term storage, artificial aging and other factors are usually accompanied by changes in the internal physiological and metabolic characteristics of the seeds (Sutton and Punja, 2017). These subtle changes affect the optical properties of the seeds. Hyperspectral imaging technology is used to detect imperceptible internal variations that are not visible to the naked eyes by capturing detailed spectral and spatial information in the visible and near-infrared spectra regions (Yu et al., 2018; Barbedo, 2023). Hyperspectral imaging is a promising technique for rapidly and non-destructive assessment seed vigor. Numerous studies have introduced HSI to capture changes in the optical properties of seeds for predicting seed vigor.

Analyzing the large amount of data generated by hyperspectral imaging presents numerous challenges. With the rapid advancement of computer vision, significant progress has been made in automating seed prediction (Nie et al., 2019; de Medeiros et al., 2020; Wang et al., 2023). Nevertheless, there are still a few urgent issues need to be addressed in predicting seed vigor using HSI.

**Firstly, there is an issue of imbalanced seed vigor samples.** The collection of seed vigor data relies on the actual germination rate, which inevitably leads to an imbalance between positive and negative samples during the collection process. Sample imbalance will result in difficulties in extracting regular features from the classes with fewer samples due to the limited number of training samples, which will easily lead to overfitting problems. Sample imbalances should be addressed without hesitation in seed vigor prediction with HSI.

**Secondly, there are differences in the wavelength extraction of HSI.** HSI typically contains hundreds of wavelengths, characterized by feature redundancy, collinearity and so on. Although more spectral features could achieve high accuracy, it might cause information redundancy and complexity. In HSI analysis, traditional machine learning algorithms have focused on improving extraction of characteristic wavelengths. Many algorithms for extracting characteristic wavelengths have been applied to HSI classification in recent years. For example, Cheng et al. (2023) used HSI in the spectral range of 400 to 1000*nm* to predict seed vigor of broad beans and hyacinth beans. Firstly, they conducted preprocessing on the data, followed by principal component analysis (PCA) and uninformative variable elimination (UVE) to select the optimal wavelengths. Simultaneously, image features were extracted from the RGB images of the three channels. Finally, random forest (RF) and support vector machine (SVM) were employed to construct classification models based on spectral data, image data, and a combination of spectral and image data. The results demonstrated that when spectral data selected by UVE was combined with image data, the SVM model achieved prediction accuracies of 91.67% and 88.89% for broad beans and hyacinth beans. However, the prediction accuracy of most traditional machine learning models based on characteristic wavelengths relies on spectral preprocessing and the selection of characteristic wavelengths, which vary with changes in the dataset and algorithm. When the dataset changes, multiple wavelength selection algorithms and analysis models need to be retried to select the most effective combination, increasing the difficulty of establishing a robust model. Deep learning has excellent self-learning capabilities, automatically extracting and learning relevant features from raw images. However, deep learning models either use all wavelengths for training or adopt non-end-to-end network structures which first employ wavelength extraction algorithms to extract characteristic wavelengths and then train deep learning. These structures limit the ability of deep learning in feature extraction and accurate classification.

Based on the aforementioned, in order to address the issue of imbalanced sample classes and extract more effective characteristic wavelengths, this research introduces the focal loss function and WAResNet network to construct an end-to-end seed vigor prediction model called Focal-WAResNet. The Focal-WAResNet model could effectively extract the effective features among different vigor seeds and solve the problem caused by sample imbalance, thereby effectively improve the ability of seed vigor identification.

To summarize, the main contributions of this paper are as follows:

- This study uses the focal loss function to address the problem of imbalanced seed vigor and improve network performance.
- An end-to-end deep learning model called WAResNet based on HSI is constructed, which can end-to-end extract the characteristic wavelengths of HSI and perform batch and non-destructive vigor prediction for seeds.
- The recognized and state-of-the-art machine learning algorithms are compared with the proposed Focal-WAResNet. The optimal preprocessing algorithm, characteristic wavelengths extraction algorithm and classification algorithm were picked.
- The proposed Focal-WAResNet compares with advanced deep learning algorithms in seed vigor prediction. The effectiveness of Focal-WAResNet is validate through ablation experiments and visualizations using t-SNE and Grad-CAM.

## 2 Related work

Numerous studies have demonstrated that the combination of machine learning with HSI has achieved significant success in seed vigor classification. Machine learning algorithms applied to HSI classification general are divided into two categories. The first category is traditional machine learning classification algorithms, including linear discriminant analysis (LDA), partial least squares-discriminant analysis (PLS-DA), K nearest neighbors (KNN), decision trees (DT), logistic regression (LR), extreme learning machine (ELM), SVM, RF and so on (Yu et al., 2021; Long et al., 2022; Xu et al., 2022; Zhang et al., 2023). The second category is deep learning which is the subset of machine learning. Deep learning has been successful applied in many smart agricultural fields, which provides potential opportunities for its application in seed vigor prediction (Tu et al., 2021; Thakur et al., 2022; Zhang et al., 2022).

Dimensionality reduction is an essential step in traditional HSI classification. The algorithms commonly used for extracting characteristic wavelengths include competitive adaptive reweighted sampling (CARS), successive projections algorithm (SPA), PCA and UVE (Wakholi et al., 2018; Pang et al., 2021a; He et al., 2022; Jin S. et al., 2022). The swarm intelligence optimization algorithms exhibit strong search capabilities in addressing practical problems. Many studies have successfully applied swarm intelligence optimization algorithms to extract characteristic wavelengths of HSI (Chu et al., 2022). Yang et al. (2021) used traditional machine learning algorithms with HSI to predict the vigor of sugar beet seeds. They applied five preprocessing algorithms: multiplicative scatter correction (MSC), savitzky-golay (SG), standard normal variate (SNV), detrend correction (DET) and second derivative (SD), followed by SPA to extract characteristic wavelengths. The SVM model was established to predict the vigor of sugar beet seeds with full spectra or characteristic wavelengths, and the accuracy of SVM-SPA-SD was 92.32%. Fan et al. (2020) conducted preprocessing using SG, SD and SNV, followed by PCA and SPA to select the most effective wavelengths. Four machine learning methods: adaptive boosting (AdaBoost), SVM, ELM and RF were used to predict the vigor of wheat seeds. The optimal ELM-PCA model achieved a classification accuracy of 88.9%.

Deep learning effectively utilizes the spatial and spectral information of HSI and have exhibited excellent performance in seed vigor prediction (Jia et al., 2023). Jin B. et al. (2022) utilized convolutional neural network (CNN) and traditional machine learning (SVM and LR) with full spectra or characteristic wavelengths selected by PCA to identify the vigor of rice seeds. The accuracy of the CNN network was 96.88%. Pang et al. (2021b) employed 2D convolutional neural network (2DCNN) with HSI to predict the seed vigor of sophora japonica. They used particle swarm optimization (PSO) to optimize network hyperparameters. The optimal PSO-2DCNN model achieved an accuracy of 99.73%.

**Table 1** summarizes the relevant researches on predicting seed vigor by combining machine learning with HSI. It is worth noting that the models in the table either utilize full spectra for training or employ non-end-to-end network structures for seed vigor prediction.

**Table 1:** Current major methods on seed vigor prediction with HSI.

Seed	Image	Preprocessing	Wavelength Selection	Classification		Accuracy	Reference
Peanut	HSI	SG, MSC, MF	CatBoost, GBDT	XGBoost, LightGBM, SVM, RF		90.83%	(Zou et al., 2023)
Maize	NIR HSI, SWIR HSI	Normalization, SNV, MSC, SG	Full	PLS-DA		95.6%	(Ambrose et al., 2016)
Bean	HSI	SG, MSC	PCA, UVE	SVM, RF		91.67%	(Cheng et al., 2023)
Beet	HSI	FD, SD, MSC, SNV, DET, SG	Information Gain	SVM, RF, LightGBM		89%	(Zhou et al., 2020)
Sugarbeet	HSI	MSC, SNV, DET, SG, SD	SPA	SVM, RF, KNN		95.5%	(Yang et al., 2021)
Maize	HSI	WT	SD, PCA	SVM		71.31%	(Feng et al., 2018)
Wheat	NIR HSI	SNV, SG	PCA, SPA	SVM, RF, ELM, AdaBoost		88.9%	(Fan et al., 2020)
Soybean	FHSI	MSC, SNV, SG	CARS, VISSA, IRIV	SVM, AdaBoost		86%	(Zhang T. et al., 2020)
Rice	NIR HSI	FD, MSC, SG	SPA	SVM, PLS-DA, ELM		94.38%	(He et al., 2019)
Rice	HSI	None	Full	DCNN-Balanced		97.69%	(Wu et al., 2022)
Sophora japonica	HSI	None	Full	2DCNN		99.73%	(Pang et al., 2021b)
Mustard spinach	NIR HSI	None	PCA, SVM Mapping	2DCNN		90%	(Ma et al., 2020)
Maize	HSI	SG, FD, DET, SNV, MSC	UVE, SPA, IRF, IVSO	1DCNN		95.24%	(Xu et al., 2022)

FD, First Derivative; RC, Regression Coefficients; MF, Median Filtering; GBDT, Gradient Decision Tree; XGBoost, Extreme Gradient Boosting; LightGBM, Light Gradient Boosting Machine; SD, Second Derivative; DET, Detrend Correction; WT, Wavelet transform; AdaBoost, Adaptive Boosting; IRIV , Iteratively Retaining Informative Variables; VISSA, Variable Iterative Space Shrinkage Approach; IRF, Interval Random Frog; IVSO, Iteratively Variable Subset Optimization; Vis-NIR HSI, Visible Near Infrared HSI; SWIR HSI, Short-wave Infrared HSI; NIR HSI, Near-infrared HSI; FHSI, Fluorescence Hyperspectral Imaging.

## 3 Materials and methods

### 3.1 Methods

#### 3.1.1 ResNet

**Figure 1:**
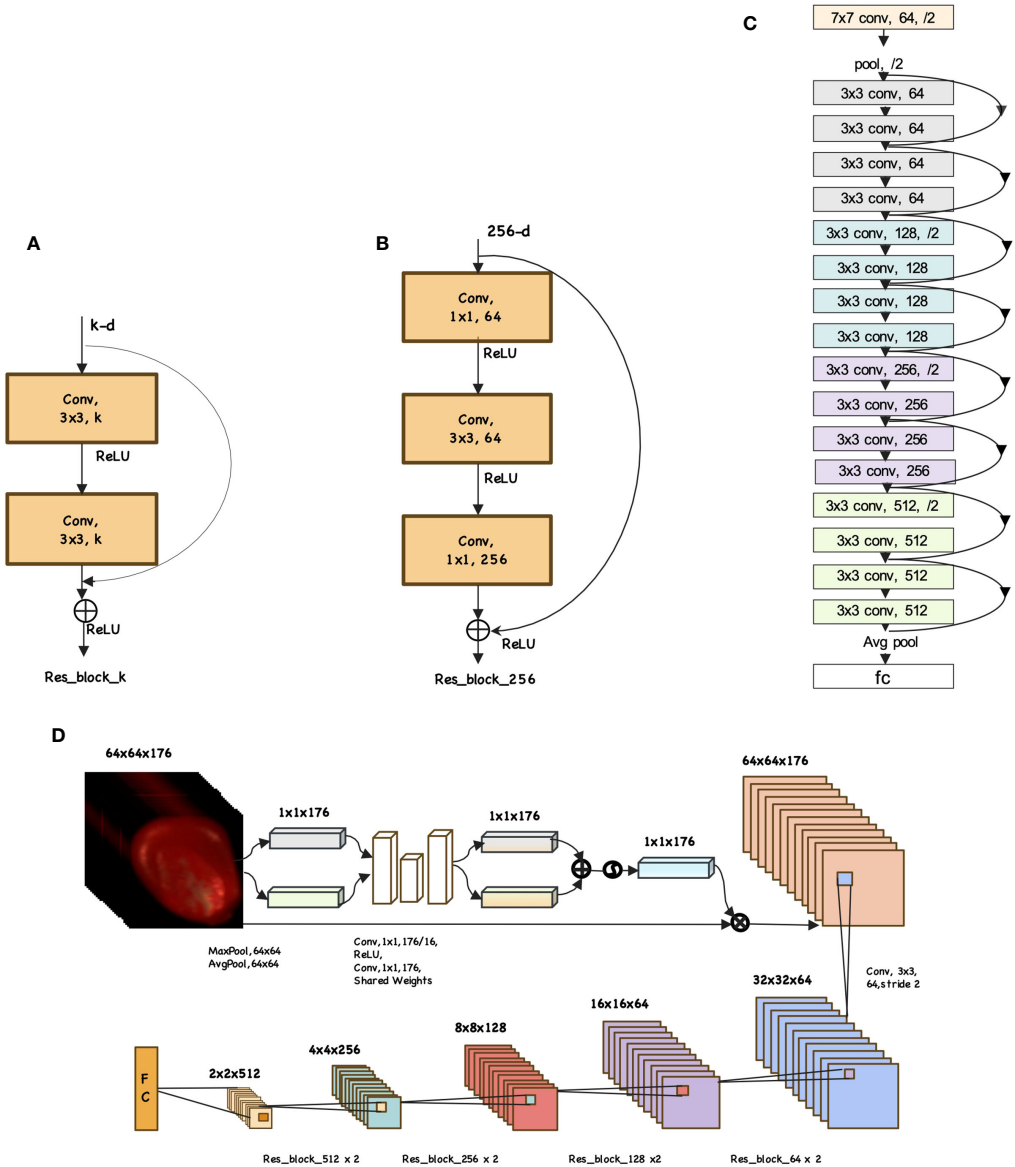
Example network architectures for mazie seed. **(A)** ResBlock. **(B)** ResBlockDeep. **(C)** ResNet18 model. **(D)** Focal-WAResNet model.

The ResNet network proposed by Microsoft Labs is champion network in both the classification and object detection tasks of the 2015 ImageNet Large Scale Visual Recognition Challenge (ILSVRC-2015) (He et al., 2016). ResNet adopts the residual structures to construct network structures. According to the number of layers, ResNet is categorized into ResNet18, ResNet34, ResNet50, ResNet101 and ResNet152. **Figure 1A** is the residual structure for ResNet with fewer layers: ResNet18 and ResNet34. **Figure 1B** depicts the residual structure for deeper network like ResNet50, ResNet101 and ResNet152. To avoid overfitting caused by high similarity among seed samples of the same variety, this study utilizes a light network architecture: ResNet18. The network architecture is depicted in **Figure 1C**. The ResNet18 consists of one stem layer, two pooling layers, eight ResBlocks and one fully-connected layer. The stem layer is a convolutional layer which kernel size is 7×7 with a stride of 2 and 64 convolutional kernels. The two pooling layers are max pooling and average pooling which kernel size is 3 with a stride of 2.

#### 3.1.2 Focal loss

Focal loss is a variant of binary cross entropy loss, which is a common loss function (Lin et al., 2017). Its formula is as follows:


(1) $$CE(p,y)=CE(p_{t})=-log(p_{t})$$

where *y* of Equation 1 is the ground-truth class of sample, and *p_t_* is the model’s estimated probability for the class. The definition of parameter *p_t_* is as indicated in Equation 2.


(2) $$p_{t}=\{\begin{array}{ll} p & y=1\\ 1-p & otherwise. \end{array}$$

Incorporate weight factor *α_t_* into Equation 1 to address the issue of class imbalance. The cross entropy loss could be expressed as Equation 3.


(3) $$CE(p,y)=CE(p_{t})=-\alpha_{t}log(p_{t})$$

where *α_t_* is defined as Equation 4.


(4) $$\alpha_{t}=\{\begin{array}{ll} \alpha & y=1\\ 1-\alpha & otherwise. \end{array}$$

While the Equation 3 could address the class imbalance problem, it does not distinguish between difficult and easy samples. A factor (1−*p_t_*)*^γ^* is introduced to the cross entropy loss. The focal loss function is defined as:


(5) $$FL(p_{t})=-\alpha_{t}{\left(1-p_{t}\right)}^{\gamma }log(p_{t})$$

Thus, Equation 5 could adjust the weights of classes and control the weights of easy and hard samples.

#### 3.1.3 Structure of Focal-WAResNet

According to the characteristic of seed HSI, we improve the ResNet18 model and propose an end-to-end model called Focal-WAResNet (wavelength attention ResNet with focal loss) for seed vigor prediction. **Figure 1D** shows the network structure of Focal-WAResNet. The network consists of two pooling layers, two 2D convolutional layers and ResNet18. The two pooling layers are max pooling and average pooling with a pooling size of 64×64 and a stride of 1. The convolutional kernel size of the two convolutional layers is 1×1, with 11 and 176 convolutional kernels respectively. The first convolution layer is followed by a ReLU activation function. Since the size of the convolutional kernel is 1×1, it only affects calculation between channels without changing the spatial resolution of the feature maps. The resolution of the HSI is normalized to 64×64 in the experiment. Given the relatively small image resolution, the stem layer of ResNet18 is replaced with a 3×3 convolutional layer with a stride of 2. At the end of this network, focal loss function is set as the loss function to measure the errors between the predicted outputs and the actual targets.

The propagation process of Focal-WAResNet network is depicted in **Figure 1D**. The input *X* ∈ **R** ^W×H×C^ passes through max pooling and average pooling layers respectively to capture the maximum feature of the channel *X_max_* ∈ **R** ^1×1×C^ and the mean feature *X_avg_* ∈ **R** ^1×1×C^. These processes could be characterized by Equations 6 and 7.


(6) $$X_{max}=MaxPooling(X)$$

(7) $$X_{avg}=AvgPooling(X)$$

The features *X_max_* and *X_avg_* separately pass through 1×1 convolution layer to reduce dimension to 11. Then, the results go through a ReLU activation function and increase the dimension to the original channel dimension *C* using another 1×1 convolution layer. These two convolutional layers share weights to reduce the number of model parameters, which can reduce model complexity and the risk of overfitting, and improve model generalization capability. These operations achieve two feature matrices and rescaling in the channel dimension. The addition of the two feature matrices passes through the *sigmoid* activation function to scale the output value to the range of 0 to 1, obtaining the wavelength attention weights *X_wa_* ∈ **R** ^1×1×C^. The formula is as Equation 8.


(8) $$X_{wa}=sigmoid(W_{1}(W_{0}X_{avg})+W_{1}(W_{0}X_{max}))$$

where *W*_0_ ∈ **R** ^C/**r**×C^ and *W*_1_ ∈**R** ^C×C/**r**^ are the weights of the two convolutional layers separately.

Finally, the wavelength attention weights are applied to each channel of the original input using Equation 9. Then the result feeds into the modified ResNet18 network to strengthen or suppress feature representations of different channels to improve the model performance.


(9) $$output=X_{wa}⊗X$$

where ⊗ is element-wise multiplication.

## 4 Results and analysis

### 4.1 Data collection

#### 4.1.1 The seed aging experiment

Since the natural aging of seeds is a prolonged process, and according to the different environments in which the seeds are located, the uncertainty of natural aging is comparatively higher. Artificial aging tests enable to artificially control the aging conditions and degree of the seeds, and get more diversified data for reference. To avoid the influence of human subjective factors on the test results, 1200 maize seeds of the same batch “Meiyu 817” were randomly selected from the seed repository before the collection of HSI. Then 600 seeds were randomly selected and divided into three groups with 200 seeds in each group. These three groups were stored at a constant temperature of 20°C, 0°C and -20°C respectively to obtain maize seeds with different degrees of aging: 20°C, 0°C and -20°C. The remaining 600 seeds were vacuum-sealed in plastic bags and placed in a water bath maintained at a temperature of 45°C and a relative humidity of 100% for aging. On the 3rd day, 6th day, and 9th day after the beginning of aging, 200 seeds were separately taken out to obtain maize seeds with different degrees of aging: 3d, 6d, 9d. After the accelerated aging process, HSI collection and standard germination tests were conducted.

#### 4.1.2 The collection of HSI

**Figure 2:**
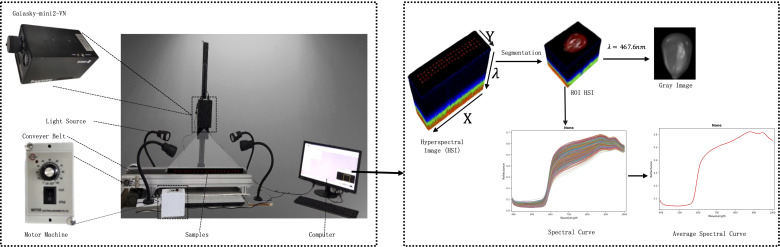
The system and process of HSI acquisition.

Obtaining high-quality HSI is the crucial step in HSI analysis. HSI combines traditional imaging with spectral information to obtain both spectral and spatial information in a single scan. In this study, the HSI system included a hyperspectral imager, lighting equipment, a conveyor belt, an electronic transmission control system, and a computer, as shown in **Figure 2**. The Gaiasky-mini2VN hyperspectral imager with the wavelength range of 393.7-1001.4*nm*, 176 spectral channels, and the single image resolution of 960 * 1040 from Dualix Spectral Imaging Technology Co., Ltd. was used in the experiment. The other parameters are shown in **Table 2**. The GaiaSky-mini2-VN constructed high-resolution images *I*(*X*, *Y*, *λ*) by scanning, where *X* and *Y* represented spatial dimensions, and *λ* represented spectral dimensions. Each pixel of HSI reflected a spectral curve, and each grayscale image corresponded to a spectral band. The lighting system consisted of four 50W halogen lamps, which needed to be adjusted to the appropriate position and warmed up for 30 minutes before the collection of HSI. During the process of capturing HSI, multiple maize seeds were placed on a blackboard. They were transported through the electronic transmission control system and conveyor belt, and photographed by Gaiasky-mini2-VN. To eliminate the effects of uneven illumination and dark current, the HSI was rectified using white and black reference images after obtaining the HSI of maize seeds. The correction formula is depicted in Equation 10.

**Table 2:** Technical parameters of GaiaSky mini2-VN.

Technical parameters of GaiaSky mini2-VN	
Device	GaiaSky mini2-VN
Wavelength range	393.7-1001.4*nm*
Spectral resolution	3.5*nm* @30*um* slit
Spectral sampling rate	0.5*nm*
Pixel pitch	4.54 *um*
Spectral channel count	176(8X)
Image resolution	960*1040
Spatial resolution	0.085@16*mm*
Power	45*W*
Voltage	DC12*V*( ± 10%)
FOV	30.25°@16*mm*

The ‘@’ symbol represents ‘at’ or ‘with’, which is used to describe measurement conditions.


(10) $$I_{o}=\frac{I-I_{b}}{I_{w}-I_{b}},$$

where *I* and *I_o_* respectively represented HSI before and after correction. *I_b_* and *I_w_* represented black and white reference images.

Finally, the region of interest which was the HSI of a single maize seed was segmented from the black background. The average spectral curve of each maize seed was extracted as shown in **Figure 2**.

#### 4.1.3 The standard germination test

Seed vigor refers to the potential germination capacity of seeds or the vitality possessed by seed embryos, representing the potential capacity of seeds to develop into healthy seedlings. In this study, we assess the vigor of maize seeds through standard germination test.

**Figure 3:**
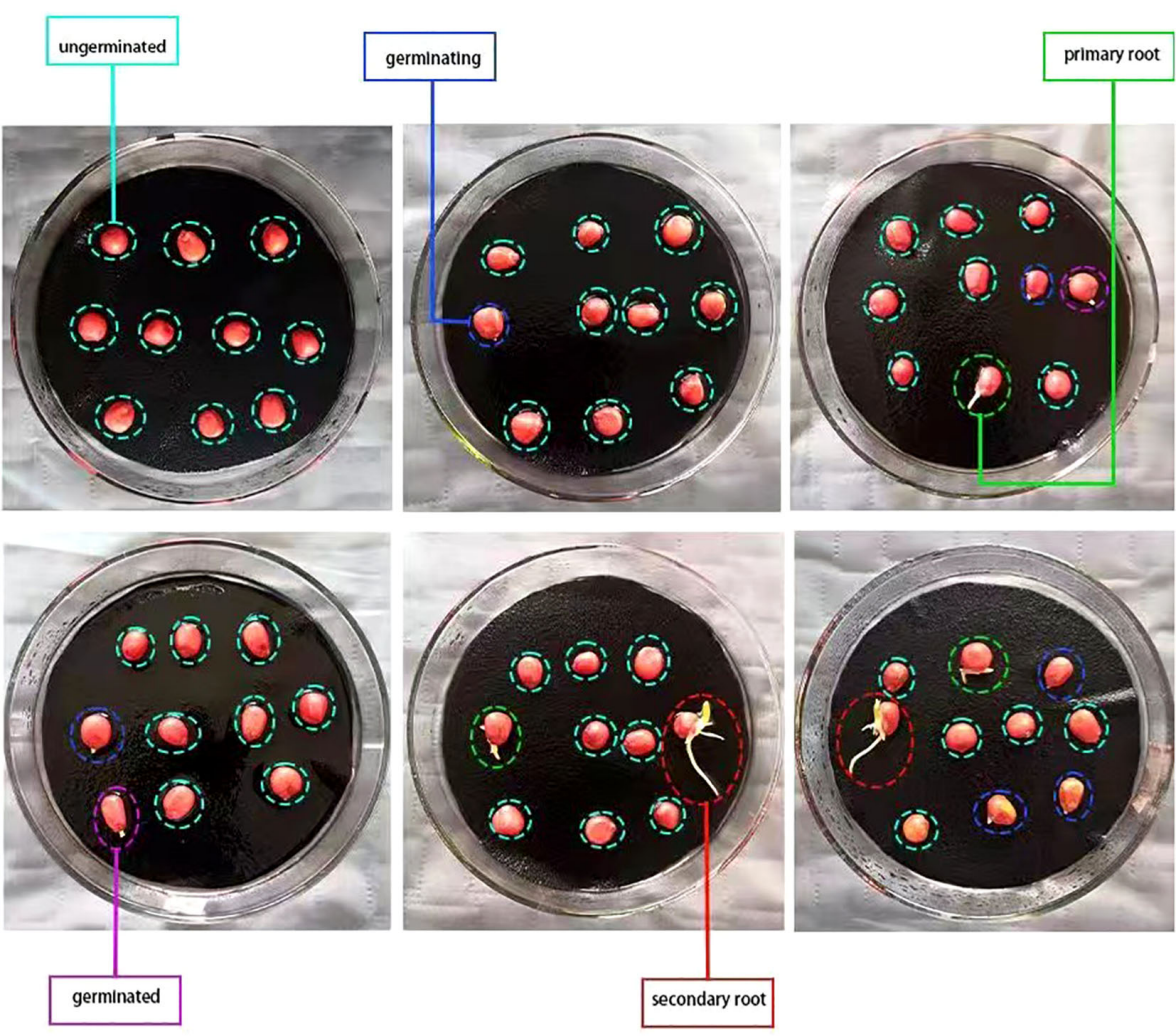
The different stages of seed germination.

According to the International Rules for Seed Testing, transparent, moisturizing and non-toxic circular petri dishes with a diameter of 120*mm* were used in the standard germination test (ISTA, 2018). Germination papers which were moistened and drain off surplus water were placed in the sterilized petri dishes. Ten seeds were evenly placed in each petri dish to ensure that each seed had a germination space with a distance of 1-2 times the seed own size, as shown in **Figure 3**. Put those petri dishes in the germination chamber. The optimum oxygen, moisture, temperature, and lighting conditions for maize seeds were provided and the germination beds remained moist throughout the germination period. According to the technical regulations for crop seed germination (GB/T 3543.4-1995), maize seeds can germinate normally and well under the optimum temperature 20-30°C. In this experiment, the thermostatic germination chamber which temperature was set at 25°C was used to ensure that the temperature variation did not exceed ±2°C.

Damaged, cracked, malformed, or uneven seeds, as well as the dead seeds which were severely decayed or moldy were promptly removed from the bud beds during the germination process and culled for counting. In the experiment, germinating seeds, germinated seeds, seeds with primary root, seeds with secondary root were defined as viable seeds, and ungerminated seeds, dead seeds and fresh ungerminated seeds were defined as non-viable seeds. As depicted in **Figure 3** that the phase in which the radicle of the seed elongates between 0-2*mm* is characterized as ‘germinating’, whereas the phase with elongation exceeding 2*mm* is characterized as ‘germinated’. The phase of generating the primary root, derived from the radicle, is termed as the ‘primary root’. The phase in which generates more than one secondary root in addition to the primary root is termed as the ‘secondary root’. On the 7th day of the standard germination test, the germination statuses of six groups of maize seeds with different degrees of aging were recorded, which was referred to as seed vigor statistics. The removed maize seeds are deleted during the statistical process, and a total of 1133 seeds are recorded, of which 915 are viable and 218 non-viable. Lastly, the dataset was divided into training set, validation set and test set at a ratio of 8:1:1.

### 4.2 Data augmentation

The total number of samples in the dataset is insufficient due to the inability to obtain a sufficient number of hyperspectral images of maize seeds during hyperspectral image acquisition and standard germination tests, which could potentially impact the classification effect of the model. In this study, online augmentation technique was employed to expand the dataset and ensure data diversity. Randomly data augmentation techniques, such as rotation, horizontal flipping, scaling, and so on, were applied during each iteration to generate different training examples. It helps the model better adapt to various input variations without the need to explicitly increase storage space to store augmented examples, reducing the risk of overfitting and improving the model’s generalization and robustness.

### 4.3 Evaluation metrics

In this study, viable seeds were regarded as positive samples, while non-viable seeds were regarded as negative samples. Accuracy, precision, recall, F1 were used to evaluate the performance of the model. The calculation formula of each metric was shown in **Table 3B**, where TP, TN, FP, and FN represent the numbers of true positive samples, true negative samples, false positive samples, and false negative samples, respectively. The corresponding confusion matrix is presented in **Table 3A**. Precision reflects the model’s ability to distinguish negative samples. A higher precision indicates a stronger ability of the model to discriminate negative samples. Recall, on the other hand, reflects the model’s ability to distinguish positive samples. A higher recall indicates a stronger ability of the model to discriminate positive samples. F1 is a combination of both precision and recall, and a higher F1 indicates a more robust model.

**Table 3:** Evaluation metrics.

(A) The definition of confusion matrix		
True Predict	Positive	Negative
Positive	TP	FP
Negative	FN	TN

(B) The calculation formula of evaluation metric		
Evaluating Indicator	Formula	
Accuracy	$Accuracy=\frac{TP+TN}{TP+FP+TN+FN}$	
Precision	$Precision=\frac{TP}{TP+FP}$	
Recall	$Recall=\frac{TP}{TP+FN}$	
F1	$F1=2\times \frac{Recall\times Precision}{Recall+Precision}$	

### 4.4 Analysis of results

#### 4.4.1 Germination and vigor statistics

**Figure 4:**
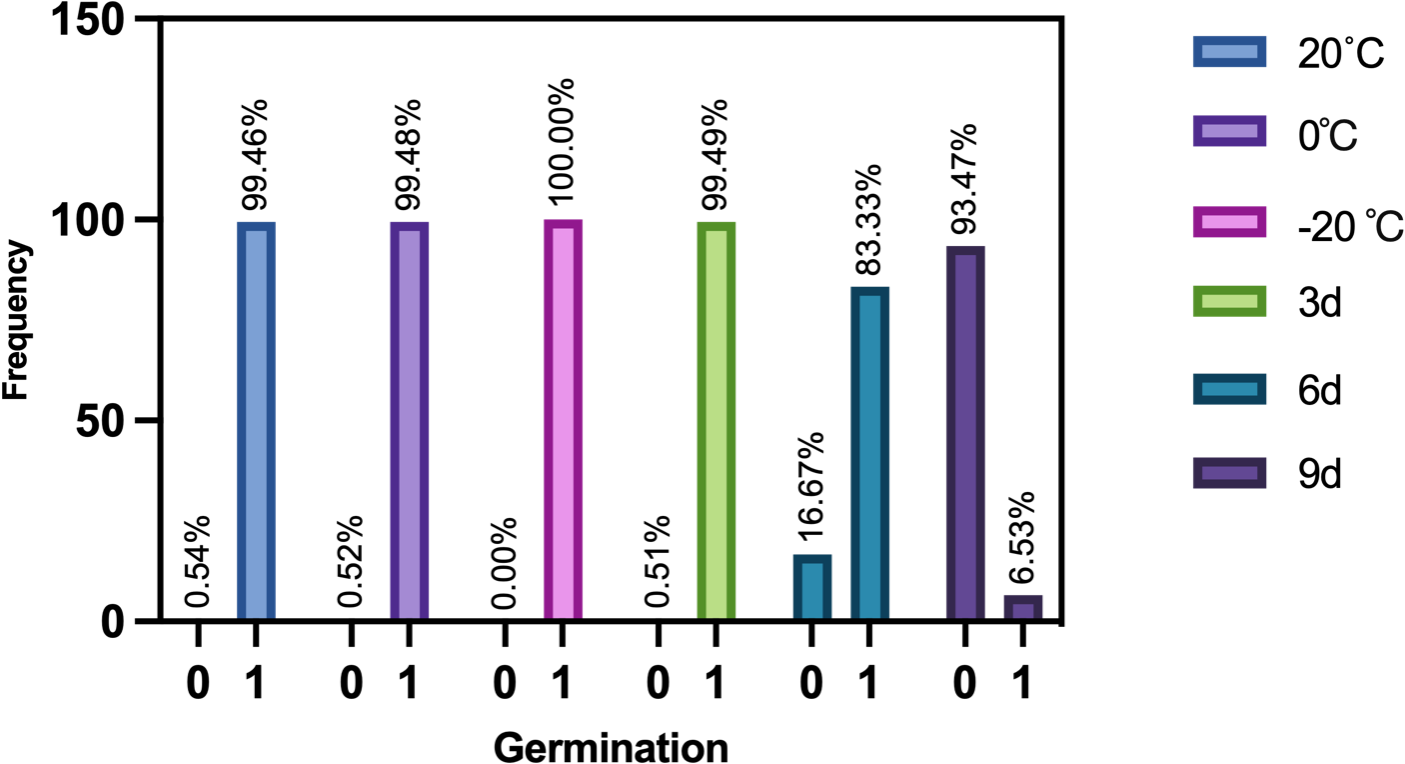
The maize seeds germination frequency at different ageing stages.

**Figure 4** is the frequency histogram representing the germination of maize seeds at different aging stages. It shows that the germination rates of maize seeds stored at 20°C, 0°C and 3 days of aging are about 99%, and the germination rate of maize seeds stored at -20°C is 100%. The germination rates of maize seeds are hardly affected in these four environments. However, the germination rate drops to 83.33% after 6 days of aging. The germination rate decreases to 6.53% after 9 days of aging. This indicates that aging for a sufficiently long period of time has a serious effect on germination and vigor.

#### 4.4.2 Spectral analysis

**Figure 5:**
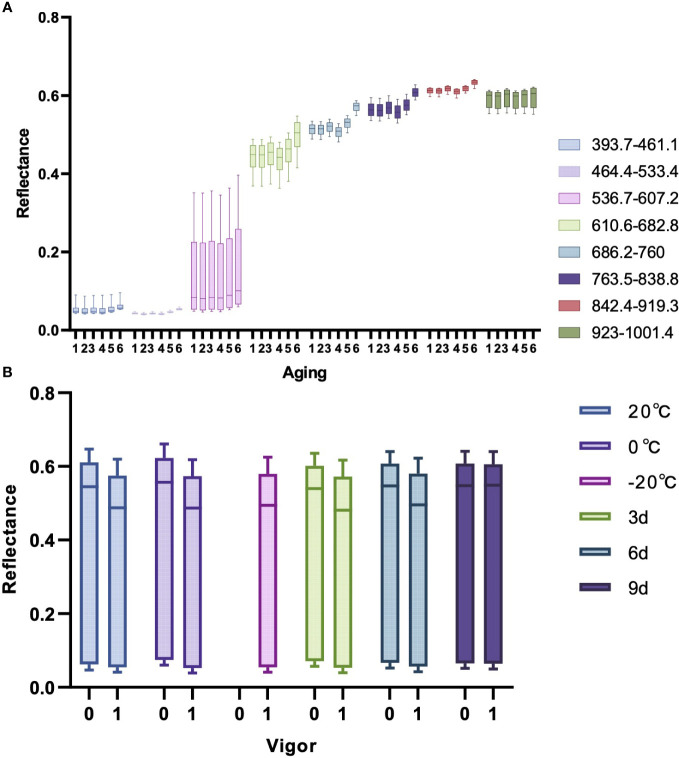
The box plots of spectral reflectance. **(A)** Box plot of spectral reflectance at different bands for maize seeds at various aging levels. **(B)** Box plot of spectral reflectance at different aging stages for seeds with different vigors.

The variations of seed vigor caused by seed aging typically accompany with changes of seed cell structure, biochemical composition and metabolic characteristic.These tiny changes can affect the optical properties of the seeds. Hyperspectral imaging technology can detect tiny changes that are invisible to the naked eyes. **Figure 5A** is a box plot of the spectral reflectance of maize seeds at different aging degrees in different bands in the experiment. Given the distinct variations in spectral reflectance across different bands, **Figure 5A** evenly divides the 176 wavelengths into eight bands to assess the impact of different degrees of aging on the spectral reflectance in different bands. Different colors in the figure represent different bands, and the values from 1 to 6 in the horizontal axis represent different aging degrees: 20°C, 0°C, -20°C, 3d, 6d, and 9d. As indicated in **Figure 5A** that there are certain differences in the average spectral reflectance of maize seeds with different aging degrees. The spectral reflectance of seeds aged for 6 days and 9 days is higher than others in each band, indicating that the more severe the degree of aging, the higher the spectral reflectance of maize seeds. The spectral reflectance of maize seeds aged for 3 days did not increase, indicating that aging for 3 days did not significantly affect the vigor of maize seeds. Therefore, the germination rate of maize seeds was also not affected, which is consistent with the results of the statistical analysis of germination rate and vigor mentioned above.

**Figure 5B** is a box plot of the spectral reflectance of maize seeds with different vigor at different aging stages. Different colors in the figure represent different aging degrees, and the values of 0 and 1 in the horizontal axis represent non-viable and viable seeds. Since the seeds stored at -20°C were all viable seeds in the experiment, there is only one box at this aging stage. As observed from the **Figure 5B** that the spectral reflectance of viable seeds stored at 20°C and 0°C, as well as maize seeds aged for 3 days and 6 days is lower than the non-viable seeds. Since the loss of vigor in maize seeds aged for 9 days has already reached the peak and the seeds will be completely non-viable if the aging continues, the spectral reflectance of the viable maize seeds aged 9 days is almost as high as that of the non-viable maize seeds. This reveals that aging of maize seeds will lead to the wastage of seed vigor and increase the spectral reflectance of maize seeds.

#### 4.4.3 Comparison with traditional machine learning

Currently, HSI classification primarily revolves around traditional machine learning algorithms. Therefore, this section provides a comparative analysis of traditional machine learning algorithms.

**Figure 6:**
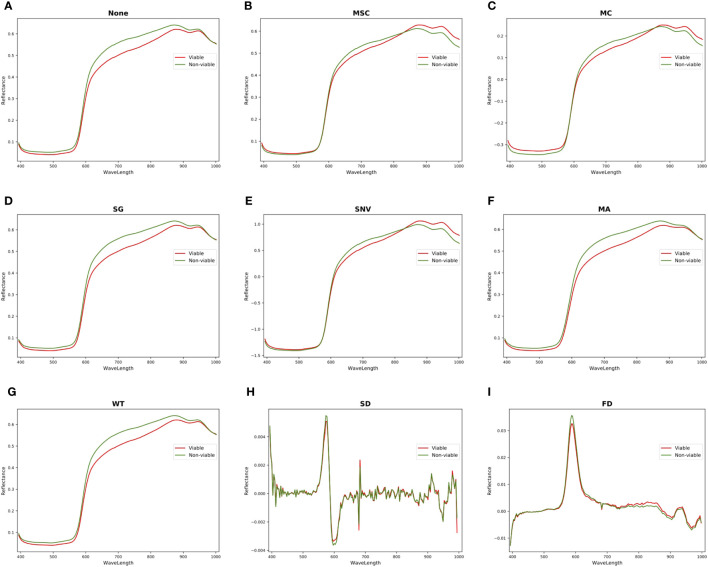
Comparison of spectral curves for viable and non-viable seeds by various preprocessing. **(A)** None. **(B)** MSC. **(C)** MC. **(D)** SG. **(E)** SNV. **(F)** MA. **(G)** WT. **(H)** SD. **(I)** FD.

The HSI obtained by hyperspectral imaging system includes various noises, such as random high-frequency noise, sample background, baseline drift, scattered light and so on. Therefore, the HSI should be preprocessed to eliminate noises before extracting characteristic wavelengths and data modeling. This experiment compared various preprocessing algorithms based on full spectra, including mean centering (MC), moving average smoothing (MA), SNV, SG, MSC, FD, SD and WT. **Figure 6** shows the comparisons of spectral curves between viable and non-viable seeds after different pretreatments with the full spectra. As depicted in **Figure 6A** that the spectral curves of viable and non-viable maize seeds exhibit similar wave patterns: peaks and valleys appear at similar band positions. This phenomenon may be attributed to the similar chemical composition within the seeds. There are significant spectral differences between viable and non-viable seeds in the wavelength ranges of 393.7-580*nm* and 620-950*nm*, which the spectral reflectance of viable maize seeds is lower than non-viable maize seeds. After MC preprocessing (**Figure 6C**), there are significant differences in spectral reflectance between viable and non-viable maize seeds in the 393.7- 580*nm* and 620-1001.4*nm* wavelength ranges. The spectral reflectance of viable seeds is higher than non-viable seeds in the 393.7-580*nm* and 880-1001.4*nm* wavelength ranges. The spectral reflectance of viable seeds is lower than non-viable seeds in the 580-880*nm* wavelength ranges. However, the spectral curves processed by MSC and SNV algorithms (**Figures 6B, E**) show an obvious overlap of spectral reflectance in the 393.7-580*nm* wavelength ranges between viable and non-viable seeds. The spectral curve processed by SD shows that the viable and non-viable seeds have more overlapping spectral reflectance. However, the spectral curves preprocessed by the SG, WT and MA algorithms do not show significant differences from the original spectral curves. Further modeling and analysis are needed to select the optimal preprocessing algorithm for predicting maize seed vigor.

**Table 4** is a statistical table of accuracy for predicting maize seed vigor using different classifiers on HSI processed by different preprocessing algorithms within the full spectra, which noticed that the MC algorithm performs the best on HSI by applying different classifiers, resulting in achieving the optimal classification accuracy of the five classification algorithms: DT, Ridge Regression, KNN, RF and PLS-DA. This is consistent with the analysis results in **Figure 6**. Because MC preprocessing increases the spectra differences between different classes, it improves the robustness and recognition ability of the model.

**Table 4:** Comparison of classification accuracy of different classifiers for seed vigor prediction (%).

Pretreat	DT	GPC	GNB	Ridge	SVM	KNN	RF	PLS-DA
None	88.99	92.95	75.33	93.39	**93.83**	91.19	91.19	92.95
**MC**	**90.31**	92.51	79.30	**93.83**	92.95	**92.07**	**93.39**	**93.83**
**MA**	86.34	**93.39**	75.77	92.95	**93.83**	**92.07**	91.63	92.95
SNV	87.22	92.07	82.82	91.63	91.63	91.19	90.31	92.51
SG	86.34	92.95	75.33	92.95	**93.83**	91.19	90.75	91.63
MSC	88.11	92.07	83.26	91.19	92.51	91.19	89.43	93.39
**FD**	88.11	92.51	**85.46**	**93.83**	92.51	84.14	91.63	**93.83**
SD	77.97	91.19	78.85	92.95	91.63	78.85	82.82	93.39
WT	89.43	92.95	75.33	93.39	**93.83**	91.19	91.19	92.95

The bold font indicates the best performances of different classifiers.

To ensure the fairness of the experiment, this study adopted the three optimal preprocessing algorithms to preprocess HSI which was used for wavelength extraction and classification analysis. As can be observed from **Table 4**, MC, MA and FD algorithms have the prominent preprocessing performance. CARS, SPA, least angel regression (LARS), UVE, PCA as well as various swarm intelligence optimization algorithms which are recognized and advanced algorithms for extracting characteristic wavelengths of HSI were used to extract characteristic wavelengths, including differential evolution (DE) (Storn and Price, 1997), grey wolf optimizer (GWO) (Mirjalili et al., 2014), PSO (Kennedy and Eberhart, 1995), whale optimization algorithm (WOA) (Mirjalili and Lewis, 2016), genetic algorithm (GA) (Holland, 1975) and bat algorithm (BA) (Yang, 2010). After characteristic wavelengths extraction, the optimal machine learning classifier is adopted for seed vigor prediction. In this study, classical machine learning classification models including gaussian process classification (GPC), gaussian naive bayes (GNB), ridge regression (Ridge), PLS-DA, SVM, KNN, RF and DT were compared. **Table 5** presents the accuracy statistics of the combined application of MC, MA, FD algorithms with characteristic wavelengths extraction algorithms and classification algorithms for predicting the vigor of maize seeds. As we could identify from **Table 5**, SVM-UVE-MA and PLS-CARS-FD models predicted the vigor of maize seeds with the highest accuracy of 95.15%.

**Table 5:** Comparing state-of-the-art machine learning methods for predicting seed vigor (%).

Pretreat	Wavelength Extraction	DT	GPC	GNB	Ridge	SVM	KNN	RF	PLS-DA
MC	None	90.31	92.51	79.30	93.83	92.95	92.07	93.39	93.83
	CARS	89.87	92.95	76.21	94.27	93.39	90.31	92.95	94.71
	SPA	89.43	93.39	76.21	92.51	93.83	90.31	91.63	92.51
	LARS	90.31	92.51	74.01	92.95	93.39	92.07	90.75	91.63
	UVE	91.19	92.95	89.43	93.83	92.95	93.39	94.27	94.27
	PCA	86.34	92.95	88.99	93.83	93.39	92.07	90.31	93.83
	DE	87.22	92.51	77.97	93.83	92.95	91.63	93.39	93.39
	GWO	89.43	92.95	86.78	94.27	92.51	93.39	94.71	93.83
	PSO	88.55	92.95	80.62	92.95	94.71	92.07	93.39	93.83
	WOA	88.55	93.39	79.74	93.39	92.95	92.95	92.51	93.39
	GA	90.31	93.39	80.62	93.83	93.83	90.75	92.51	93.83
	BA	88.55	92.95	81.94	93.39	91.63	90.75	92.95	92.95
FD	None	88.11	92.51	85.46	93.83	92.51	84.14	91.63	93.83
	**CARS**	88.11	93.83	87.22	94.71	92.95	88.99	92.95	**95.15**
	SPA	88.11	93.83	90.31	94.27	92.51	88.99	92.07	94.27
	LARS	88.11	92.07	89.87	91.63	92.95	88.55	90.75	91.63
	UVE	88.55	92.51	88.55	93.83	93.39	87.67	92.07	93.83
	PCA	83.70	87.67	88.55	92.07	92.51	85.90	88.99	92.51
	DE	85.46	92.07	88.55	92.51	92.51	91.63	92.51	92.51
	GWO	86.34	92.95	89.43	91.19	92.51	92.95	91.63	92.95
	PSO	88.11	92.51	87.67	94.27	93.39	89.43	92.51	93.83
	WOA	88.99	91.63	91.63	88.55	90.75	88.11	92.51	88.11
	GA	86.78	92.51	83.26	93.39	93.39	86.34	92.07	94.27
	BA	83.26	92.95	87.67	93.39	91.63	91.19	90.31	92.95
MA	None	86.34	93.39	75.77	92.95	93.83	92.07	91.63	92.95
	CARS	86.34	92.95	77.09	92.51	94.27	92.07	91.63	94.27
	SPA	88.99	92.95	72.69	92.07	93.39	92.07	92.07	92.07
	LARS	85.02	92.07	73.13	92.51	92.07	90.75	91.63	93.83
	**UVE**	88.11	93.39	72.25	93.83	**95.15**	91.63	94.27	94.27
	PCA	83.26	93.39	89.43	92.95	93.39	92.07	89.43	93.83
	DE	85.90	92.07	77.09	92.95	93.39	93.39	91.19	92.95
	GWO	86.78	92.51	76.21	92.51	92.51	92.07	91.63	92.07
	PSO	87.67	92.95	73.13	93.39	93.39	91.63	92.07	92.95
	WOA	86.34	92.51	77.09	92.51	92.07	92.07	90.31	91.63
	GA	87.22	92.95	74.89	93.39	93.83	91.19	92.07	93.83
	BA	87.22	93.39	75.77	93.39	93.83	91.63	92.07	92.51

The bold font indicates the best performances.

#### 4.4.4 Comparison with deep learning classifiers

The Focal-WAResNet proposed in this article was compared with advanced deep learning researches. The comparison primarily focused on two aspects: one is the comparison with nonend-to-end network architecture (NETE), the other is the comparison with end-to-end network architecture (ETE). As shown in **Table 6**, the Focal-WAResNet outperforms previous researches in performance. The accuracy of Focal-WAResNet surpasses the state-of-the-art non-end-to-end network PCA-1DCNN by 1.565%. Compared with end-to-end network architectures, it achieves an accuracy improvement of 1.044%.

**Table 6:** Comparing with the deep learning model (%).

Type	Model	Reference	Accuracy
NETE	PCA+SVM-2DCNN	(Ma et al., 2020)	90.10
	CARS-1DCNN	(Zhang C. et al., 2020)	91.67
	SPA-1DCNN		95.83
	PCA-1DCNN		96.88
	WT-1DCNN		95.31
ETE	DCNN-Balanced	(Wu et al., 2022)	97.40
	2DCNN	(Pang et al., 2021b)	94.79
	**Our**	–	**98.44**

The bold font indicates the best performances.

#### 4.4.5 Ablation experiment

In this section, we conducted ablation experiments on the maize HSI to validate the effectiveness of the focal loss function and the WAResNet network, and better understand the proposed method. We utilize t-SNE (t-distributed stochastic neighbor embedding) and Grad-CAM visualization tools to enhance our comprehension of the proposed model. t-SNE serves as a technique for non-linear dimensionality reduction and visualization of high-dimensional data (Van der Maaten and Hinton, 2008). It calculates the similarity between samples in high-dimensional space through gaussian joint probabilities, and constructs a similar probability distribution in low-dimensional space. It employs KL divergence to measure the difference between these two probability distributions, and minimizes this difference through optimization algorithms like gradient descent. The relationships and clustering among data points after dimensionality reduction could be observed more straightforward. Grad-CAM generates a heatmap by computing the gradients of the output feature map of a convolutional layer with respect to a specific class (Selvaraju et al., 2017). The heatmap contributes to understanding the image regions of that the model focuses on, providing interpretability into the model’s decision-making process.

**Figure 7:**
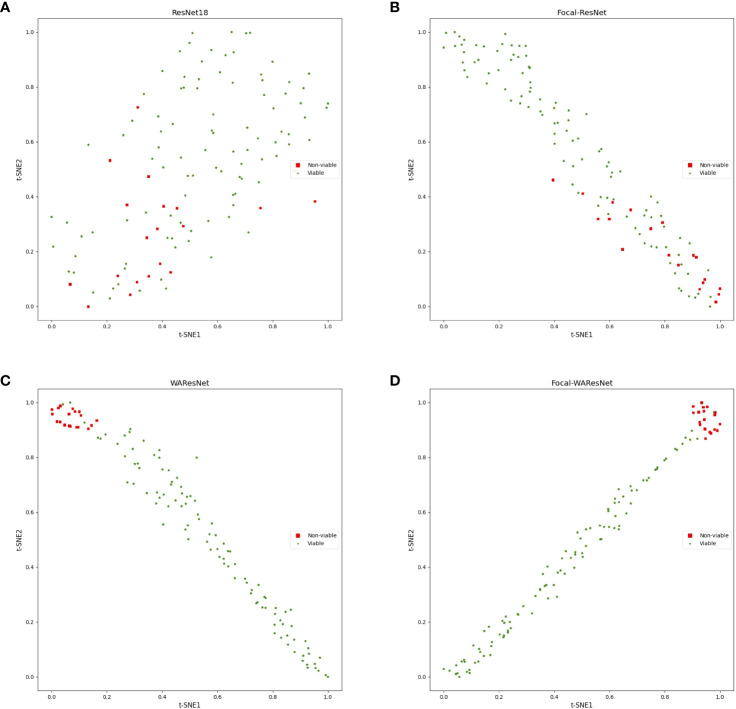
t-SNE visualization of the last layer of features of models. **(A)** ResNet18. **(B)** Focal-ResNet. **(C)** WAResNet. **(D)** Focal-WAResNet.

In the experiment, ResNet18 was used as the baseline. Firstly, the loss function of ResNet was replaced with focal loss function, which was expressed as Focal-ResNet. As shown in **Table 7**, although the accuracy of Focal-ResNet is only 1.05% higher than ResNet18 network, the precision improves by 31.38%, recall increases by 14.63%, and F1 score increases by 20.9%. It can be concluded that focal loss effectively addresses the issue of imbalanced samples. To better observe the impact of the focal loss function on model performance, this study utilized t-SNE technique to visualize the features from the last layer of the deep learning models in two-dimensional space. As depicted in **Figures 7A, B** that the feature distribution between viable and non-viable seeds of the last layer of the ResNet18 is relatively scattered and with no obvious distinction. However, the Focal-ResNet network is able to distinguish between viable and non-viable seeds.

**Table 7:** Ablation experiments for the Focal-WAResNet model (%).

Model	Accuracy	Precision	Recall	F1
ResNet18	90.10	45.05	50.00	47.40
Focal-ResNet	91.15	76.43	64.63	68.30
WAResNet	94.79	82.76	97.11	88.10
**Our**	**98.44**	**93.18**	**99.13**	**95.90**

The bold font indicates the best performances.

**Figure 8:**
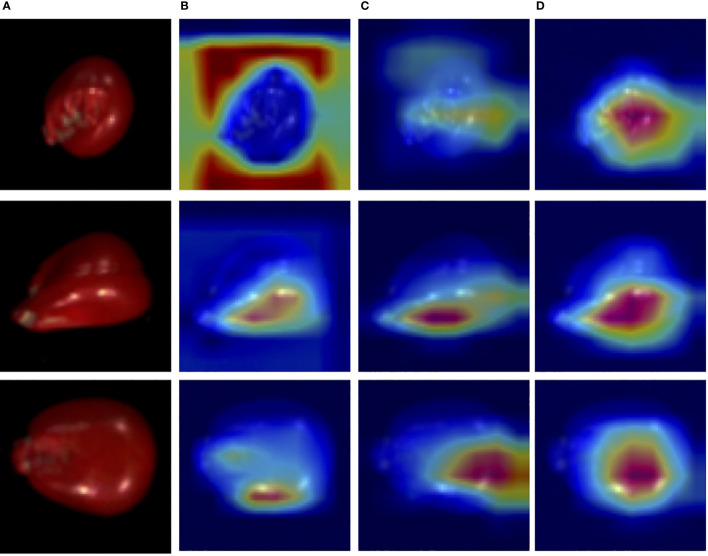
The three-channel heatmaps of maize seeds from Focal-WAResNet. **(A)** RGB. **(B)** Focal-ResNet. **(C)** WAResNet. **(D)** Focal-WAResNet.

Then, the performance of the WAResNet network has been evaluated. As shown in **Table 7**, the accuracy, precision, recall and F1 score increase by 4.69%, 37.71%, 47.11% and 40.7% respectively. **Figure 8** displays three-channel heatmaps of different models. The red regions in the heatmap represent strongly activated regions of the network model, while the blue regions represent weakly activated regions of the network model. The steeper the gradient, the redder the region, indicating that the region has a greater impact on the classification results. It is evidenced from **Figure 8C** that the WAResNet network is able to significantly focus on the region associated with seed vigor in HSI and extract features compared to Focal-ResNet.

Finally, WAResNet network is combined with the focal loss function to form the Focal-WAResNet network. The accuracy of the Focal-WAResNet network increases to 98.44%, precision to 93.18%, recall to 99.13%, and F1 to 95.90%. It can be observed from **Figure 8D** that Focal-WAResNet network can better extract features and locate the key locations which are related to seed vigor compared to Focal-ResNet and Focal-WAResNet. Meanwhile, as revealed in **Figure 7** that Focal-WAResNet might gradually make the features of maize seeds distinguishable. Samples within the same category are closely clustered, while samples between different categories become discrete, making the samples from the original cross-mixed state into a more clearly discernible state. The experiments have demonstrated that Focal-WAResNet network could effectively end-to-end extract characteristic wavelengths from HSI, allowing it to learn subtle differences between different vigor seeds. This provides new insights for seed vigor prediction.

## 5 Conclusion

This paper proposes a deep learning network structure called Focal-WAResNet, which combines deep learning algorithms with HSI to predict seed vigor. The proposed method employed the focal loss function to adjust the loss weights for different classes, thereby resolving the problem of imbalanced seed vigor samples. WAResNet achieves characteristic wavelengths and classification in an end-to-end manner by adjusting the weights of different channels to enhance or suppress the feature representation of different channels in the channel dimension. Experimental results demonstrate that Focal-WAResNet can effectively locate the regions relevant to seed vigor to achieve characteristic wavelengths of HSI and non-destructive seed vigor prediction under imbalanced sample conditions. The model could also be utilized to predict the vigor of other plant seeds. In future research, we will acquire more informative and multidimensional data to further enable seed vigor classification into no vigor, low vigor, medium vigor, and high vigor. In addition, we will explore solutions for labeling noise, multiscale and multimodal data fusion in seed vigor prediction to further improve the performance of model.

## Data availability statement

The datasets presented in this article are not readily available because the dataset is private. Requests to access the datasets should be directed to Pangtt18@mails.jlu.edu.cn.

## Author contributions

TP: Data curation, Investigation, Software, Visualization, Writing – original draft, Writing – review & editing. CC: Data curation, Formal analysis, Methodology, Writing – review & editing. RF: Methodology, Writing – review & editing. XW: Funding acquisition, Supervision, Writing – review & editing. HY: Conceptualization, Formal analysis, Methodology, Writing – review & editing.
